# Determinants of sputum smear nonconversion in smear-positive pulmonary tuberculosis patients in Suriname, 2010 – 2015

**DOI:** 10.26633/RPSP.2019.86

**Published:** 2019-12-20

**Authors:** Eric Commiesie, Deborah Stijnberg, Diana Marín, Freddy Perez, Mauro Sanchez

**Affiliations:** 1 Ministry of Health Paramaribo Suriname Ministry of Health, Paramaribo, Suriname.; 2 Universidad Pontificia Bolivariana Medellín Colombia Universidad Pontificia Bolivariana, Medellín, Colombia.; 3 Department of Communicable Diseases and Environmental Determinants of Health Pan American Health Organization/World Health Organization Washington, DC United States of America Department of Communicable Diseases and Environmental Determinants of Health, Pan American Health Organization/World Health Organization, Washington, DC, United States of America.; 4 Universidade de Brasilia Brasilia Brazil Universidade de Brasilia, Brasilia, Brazil.

**Keywords:** Tuberculosis, pulmonary, treatment outcome, sputum, risk factors, tuberculosis, Suriname, Tuberculosis pulmonar, resultado del tratamiento, esputo, factores de riesgo, tuberculosis, Suriname, Tuberculose pulmonar, resultado do tratamento, escarro, fatores de risco, tuberculose, Suriname

## Abstract

**Objective.:**

To identify factors associated with sputum smear nonconversion in patients with pulmonary tuberculosis (PTB) in Suriname.

**Methods.:**

A case-control study was conducted using routinely-collected surveillance data of PTB cases reported in January 2010 – December 2015 and recorded in the database of the National Tuberculosis Program of Suriname. Cases were smear-positive PTB patients whose sputum results were negative 2 months after treatment initiation. Controls were the smear-positive PTB patients whose sputum results were negative in the same timeframe. Multivariate logistic regression analysis was used to examine associations between potential risk factors and smear conversion.

**Results.:**

The two age groups ≥ 35 years (35 – 54 years, AOR: 2.7, 95%CI: 1.2 – 6.1; and 55+ years, AOR: 2.5, 95%CI: 1.1 – 5.9) and high bacillary load at baseline (AOR 2.34, 95%CI: 1.2 – 4.8) were significantly associated with delayed smear conversion.

**Conclusion.:**

The National TB program of Suriname should develop strategies to address patients at higher risk for delayed smear conversion to prevent further spreading and unfavorable treatment outcomes. To better inform decision-making and future studies, the NTP should expand its data collection to include all risk factors for delayed smear conversion.

## Abstract (es)

**Objetivo.:**

Determinar los factores relacionados con la falta de conversión de la baciloscopia en pacientes con tuberculosis pulmonar (TBP) en Suriname.

**Métodos.:**

Se llevó a cabo un estudio de casos y testigos con datos de vigilancia sistemática de la base de datos del programa nacional contra la tuberculosis de Suriname, se analizaron los casos de TBP notificados desde enero del 2010 hasta diciembre del 2015. Los casos fueron pacientes con TBP y baciloscopia positiva inicial cuyo resultado se volvió negativo al cabo de 2 meses del inicio del tratamiento. Los controles fueron pacientes con TBP y baciloscopia positiva inicial que presentaron resultados negativos de la baciloscopia durante el mismo período. Se usó un análisis de regresión logística multivariante para examinar las asociaciones entre los posibles factores de riesgo y la conversión de la baciloscopia.

**Resultados.:**

Los grupos etarios de mayores de 35 años (35-54 años, ORA, 2,7, IC 95 %: 1,2-6,1; y mayores de 55 años, ORA, 2,5, IC 95 %: 1,1-5,9) con carga bacilar elevada al inicio del estudio (ORA, 2,34, IC 95 %: 1,2-4,8) presentaron una asociación significativa con retraso de la conversión de la baciloscopia.

**Conclusiones.:**

El programa nacional contra la tuberculosis de Suriname debe plantear estrategias para atender a los pacientes con un riesgo más alto de retraso de la conversión de la baciloscopia a fin de prevenir una mayor propagación de la enfermedad y resultados desfavorables del tratamiento. Este programa debe ampliar la recopilación de datos para incluir todos los factores de riesgo de retraso de la conversión de la baciloscopia y de esta manera fundamentar mejor la toma de decisiones y los estudios futuros.

## Abstract (pt)

**Objetivo.:**

Identificar os fatores associados à não conversão da baciloscopia de escarro em pacientes com tuberculose pulmonar no Suriname.

**Métodos.:**

Estudo de caso-controle conduzido baseado em dados de vigilância coletados rotineiramente de casos de tuberculose pulmonar notificados em janeiro de 2010 a dezembro de 2015 e registrados na base de dados do Programa Nacional de Combate à Tuberculose do Suriname. Os casos foram pacientes com tuberculose pulmonar com baciloscopia positiva cujos resultados ficaram negativos em dois meses após o início de tratamento. O grupo de controle foi formado por pacientes com tuberculose pulmonar e baciloscopia positiva inicial cujos resultados foram negativos no mesmo período. Uma análise de regressão logística multivariada foi realizada para examinar as associações entre os fatores de risco em potencial e a conversão da baciloscopia.

**Resultados.:**

Idade de 35 anos ou mais (35 a 54 anos, OR ajustado 2,7, IC 95% 1,2–6.1; e 55 anos ou mais, OR ajustado 2,5, IC 95% 1,1–5,9) e alta carga bacilar inicial (OR ajustado 2,34, IC 95% 1,2–4,8) tiveram associação significativa com a conversão tardia da baciloscopia.

**Conclusões.:**

O Programa Nacional de Combate à Tuberculose do Suriname deve elaborar estratégias para contemplar os pacientes com maior risco de conversão tardia da baciloscopia para evitar propagar mais a doença e prevenir desfechos desfavoráveis no tratamento. Para subsidiar melhor a tomada de decisão e estudos futuros, o Programa de Tuberculose deve expandir a coleta de dados para abranger os fatores de risco para conversão tardia da baciloscopia.

Tuberculosis (TB) continues to be a global health problem with an estimated 10 million cases annually. According to the Global Tuberculosis Report 2017, in the past 5 years TB has caused more deaths than any other single infectious disease, including HIV/AIDS (1). The TB situation challenges the 2030 Sustainable Development Goal targets of reducing TB deaths by 90% and the TB incidence rate by 80% (2). According to the Global Tuberculosis Report 2017, there were an estimated 10.4 million patients with TB in 2016, of which 90% were adults, 65% male, and 10% people living with HIV (1). In 2016, new TB cases in the Region of the Americas were estimated at 274 000. Of these, 140 (0.05%) were reported by Suriname. In the same year, both the Region of the Americas and Suriname had treatment success rates close to 76% for new and relapsed cases (1, 3).

The Republic of Suriname is located on the northeast coast of South America, bordered by the Atlantic Ocean to the north, Brazil to the south, French Guiana to the east, and Guyana to the west. The country has a total land area of 163 820 km^2^. The coastal area comprises two urban and six rural districts; the interior has two districts. The two urban districts, Paramaribo (the capital) and Wanica, cover 0.5% of the country’s land and contain 70% of its population (4), which in 2017 was estimated to be 563 402 (5). The estimated TB incidence rate was 29 per 100 000 population; for TB/HIV, 4.7; and for rifampicin/multidrug resistance, 2.0 (3).

The National Tuberculosis Program of Suriname (NTP), within the Bureau of Public Health of the Ministry of Health, is responsible for TB policy development, supervision, treatment, patient follow-up, and monitoring and evaluation. TB case finding is passive in Suriname. Diagnosis is established through sputum smear microscopy and chest radiography for pulmonary TB (PTB) and other systemic investigations for extra-pulmonary disease. Xpert® MTB/RIF assay (Cepheid, Sunnyvale, California, United States; Xpert) has been used as a diagnostic tool since 2012 in Suriname.

All TB diagnostic services and treatment are free of charge in Suriname. Patients are classified according to WHO definitions and the reporting framework for tuberculosis (6). Presumptive TB patients can be referred by a family doctor to the lung clinics of the Academic Hospital Suriname or can use NTP walk-in service. In the interior of the country, patients can visit the Medical Mission; and in remote parts of the coastal area, the Regional Health Service (RGD).

Patients with delayed smear conversion 2 months after treatment initiation remain potentially infectious and are more likely to have unfavorable treatment outcomes (7). Pefura and colleagues stated that 5% – 30% of patients with a first episode of smear-positive PTB remain positive after 2 months of treatment (8). In patients with PTB, shortening the time to sputum conversion is desirable to reduce the likelihood of mycobacterial transmission.

Several factors, such as male sex, older age, higher baseline sputum grade, smoking habits, extensive disease involvement on chest x-ray (7, 9 – 13), and diabetes mellitus (14 – 17) have been identified in previous studies as risk factors for delayed smear conversion. On the other hand, Saffari and colleagues (18) found no association between delayed smear conversion and addictions, alcoholism, diabetes mellitus, or sex. The association between HIV status and smear conversion has not been fully understood. Senkoro and colleagues (15) demonstrated that HIV status did not influence sputum smear conversion. On the other hand, Kayigamba and colleagues (14) and Mlotshwa and colleagues (19) highlighted that HIV was an important predictor of sputum smear nonconversion among smear-positive PTB patients.

Predictors of smear nonconversion at baseline can help identify cases at risk for failure of TB treatment. To date, no such study has been conducted in Suriname. Therefore, the objective of this study was to identify factors associated with sputum smear nonconversion in PTB patients in Suriname in order to better guide national policy and clinical management of patients.

## MATERIALS AND METHODS

### Study design

This was a case-control study based on surveillance data collected in 2010 – 2015 by the NTP of Suriname. The study included all new and previously-treated, smear-positive PTB patients who were diagnosed in 2010 – 2015 and whose cases were notified to NTP and registered in the electronic National TB database. A TB register was used to complete the dataset with data regarding the degree of sputum smear positivity.

Patients < 15 years of age with no sputum smear result 2 months after treatment initiation, not treated, or lost to follow-up before 2 months (including patients who died) were excluded from the study. Also excluded were non-pulmonary patients, smear-negative PTB patients, and patients with unknown sputum smear status at treatment initiation.

#### Cases and control definitions.

A case of sputum smear nonconversion was defined as a patient with PTB, who had a positive smear at treatment initiation and remained positive after 2 months of treatment. A control was defined as the same except that the smear was negative after 2 months of treatment.

#### Diagnosis.

The Medical Mission (in the interior) and the RGD (coastal area) collect sputum in presumptive TB cases and do treatment follow-up in their respective service areas. TB microscopy diagnosis is performed centrally, in Paramaribo, at the Central Laboratory of the Bureau of Public Health or at the Laboratory of the Academic Hospital. Both laboratories used Ziehl Nielsen for staining and perform Xpert. Only the Central Laboratory performs TB culture. TB patients are routinely admitted and treated in the Sanatorium (Academic Hospital TB ward) until they are smear negative.

### Variables and data collection

The following data were collected for all patients: year of registration; age at time of diagnosis; sex; time (in days) since starting treatment (calculated by subtracting date of TB treatment initiation from date of admission/first positive smear); grade of smear positivity at baseline (≤1+, 2+ and ≥2+); HIV status (negative/positive/unknown); anti-retroviral therapy (ART; yes/no/ unknown); Directly Observed Treatment (yes/no/unknown); hospitalization (yes/ no/unknown); days of hospitalization; case type (new/retreatment); rifampicin sensitivity (resistant/sensitive/unknown); and comorbidity with diabetes mellitus (DM).

Among these variables, the label “unknown” refers to missing data. Factors such as being homeless, smoking, being incarcerated (or < 2 years out of prison), and alcohol and drug use were categorized together as “other risk factor” in 2010 – 2011, but as individual factors in 2012 – 2015. Therefore, in order to analyze the entire study period, the individual factors were re-coded as “other risk factor.” However, many of the “other risk factor” variables were categorized as “unknown” (missing), which could have resulted in a bias if all cases with missing data were excluded (178 of 469 cases). To resolve the issue, the association between this variable and others, as well as its effect on the model, were evaluated. It was determined that there was no significant improvement or change in the model explanation capability, so “other risk factor” was eliminated from the analysis.

The criterion “hospitalized” was defined as a hospital stay of ≥ 14 days. Several studies indicate that after treatment with multiple drugs, TB cases become smear negative in 2 – 3 weeks (13). Cases not admitted or admitted for < 14 days were classified as “not hospitalized.”

### Analysis

Data were exported from Microsoft Access™ to a Microsoft Excel™ database (Microsoft Corp., Redmond, Washington, United States) and statistical analysis was performed using IBM SPSS® Statistics, version 22 (IBM Corp., Armonk, New York, United States) and Stata® 11.1 (StataCorp, College Station, Texas, United States). The qualitative variables were described as absolute and relative frequencies; missing or unknown values were retained in the descriptive analysis. Age, time of hospitalization, and time to the start of TB treatment were reported as a median with interquartile range (IQR) because they did not follow a normal distribution. Differences between the cases and controls were assessed using the chi-square test, Fishers exact test, and the likelihood ratio for qualitative variables. The Mann–Whitney U test was used to compare medians.

An extension of the Wilcoxon rank-sum test was used to determine any significant trend in the proportion of sputum smear nonconversion in the cohort of smear-positive PTB patients. Smear status at month 2 was categorized as a binary variable. A bivariate analysis to estimate the Odds Ratio (OR) and the 95% Confidence Intervals (95%CI) was performed. The multivariate logistic regression included those variables that attained a *P* value < 0.25 in the bivariate analysis and HIV status. HIV was forced into the model because several studies describe it as a significant predictor for delayed smear conversion and it is of major concern in the TB epidemiology of Suriname (18.6% of the study population was HIV-positive). A two-tailed *P* value < 0.05 was considered significant.

### Ethics

Ethics approval for this study was obtained from the Human Scientific Research Ethic Committee of the Ministry of Health of Suriname and the PAHO Ethics Committee. Researchers had written permission to use information from the NTP. Identifiable data were removed and not used in data analysis.

## RESULTS

**FIGURE 1.**
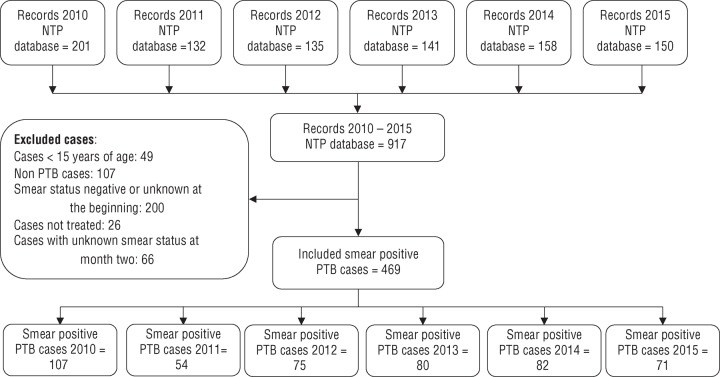
Flow diagram of cases of pulmonary tuberculosis (PTB) analyzed, National Tuberculosis Program (NTP) database, Suriname, 2010 – 2015

***Source:*** Prepared by the authors from the study results.

Of 917 records in the NTP database, 469 cases of smear-positive PTB met the study criteria and were included in the analysis (Figure 1).

The median time from diagnosis to the start of treatment was 1 day for cases (IQR = 0 – 5) versus 4 days for controls (IQR = 0 – 8). This difference was statistically significant (*P* = 0.015).

Of all the PTB patients, 12.3% remained smear-positive two months after treatment initiation. No trend in the rate of smear nonconversion was identified in the study period (2010 – 2015). Culture results were available for 89% of cases and 80% of controls. Of the controls, 64% were culture positive and identified as *Mycobacterium tuberculosis*. Of those with delayed sputum conversion and a culture result available, 94% had a positive culture at treatment initiation versus 64% of those whose smear was negative at month 2. Of the positive cultures, 96% were identified as *Mycobacterium tuberculosis* by Xpert.

Not all patients had a rifampicin sensitivity result because Xpert was not implemented until 2012 and not all were tested consistently thereafter.

The multivariate analysis (Table 2) showed that the following factors were significant predictors of delayed smear conversion after adjusting for other factors: age group 35 – 54 years (AOR: 2.7, 95%CI: 1.2 – 6.1), age group 55+ years (AOR: 2.5, 95%CI: 1.1 – 5.9), and high bacillary load (3+; AOR 2.34, 95%CI: 1.2 – 4.8).

**TABLE 1.** Demographic, clinical, and programmatic characteristics of smear-positive pulmonary tuberculosis (PTB) patients, Suriname, 2010 – 2015

Characteristic	Total (*n* = 469)	%	Cases (*n* = 59)	%	Controls (*n* = 410)	%	*P* value
Sex							
Male	349	74.4	47	79.7	302	73.7	0.323^a^
Female	120	25.6	12	20.3	108	26.3	
Age (years)							
Median	42		43 (37 – 54)		42 (31 – 51)		0.126^a^
Age groups (years)							
15-34	158	33.7	12	20.3	146	35.6	0.036^a^
35-54	240	51.2	39	66.1	201	49.0	
55+	71	15.1	8	13.6	63	15.4	
HIV status							
Negative	371	79.1	45	76.3	326	79.5	0.413^b^
Positive	87	18.6	11	18.6	76	18.5	
Unknown	11	2.3	3	5.1	8	2.0	
Receiving ART							
Yes	66	75.9	10	90.9	56	73.7	0.241^b^
No	13	14.9	1	9.1	12	15.8	
Unknown	8	9.2	0	0.0	8	10.5	
DOT							
Yes	309	65.9	41	69.5	268	65.4	0.532^a^
No	160	34.1	18	30.5	142	34.6	
Hospitalization							
Yes	417	88.9	53	89.8	364	88.8	0.898^b^
No	15	3.2	2	3.4	13	3.2	
Unknown	37	7.9	4	6.8	33	8.1	
Hospitalization (days)							
Median (IQR)	48 (30 – 71)		93 (54 – 132)		45 (29 – 66)		< 0.001^c^
Case type							
New	430	91.7	53	89.8	377	92.0	
Retreatment	39	8.3	6	10.2	33	8.0	0.612^d^
Diabetes Mellitus							
Yes	65	13.9	5	8.5	60	14.6	0.200^a^
No	404	86.1	54	91.5	350	85.4	
Bacillary load							
≤1+	196	41.8	15	25.4	181	44.1	0.015^a^
2+	103	22.0	14	23.7	89	21.7	
3+	170	36.2	30	50.8	140	34.1	
Time to start TB treatment (days)							
Median (IQR)	3 (0 – 8)		1 (0 – 5)		4		0.015^c^
Rifampicin sensitivity^e^							
Resistant	27	5.8	4	6.8	23	5.6	0.398^b^
Sensitive	237	50.5	34	57.6	203	49.5	
Unknown	205	43.7	21	35.6	184	44.9	

***Note:*** HIV = Human Immunodeficiency Virus; ART = Antiretroviral Therapy; IQR = interquartile range; DOT = Directly Observed Treatment. Other risk factors included smoking, alcohol, homelessness, prison, and drug use. Hospitalization was defined as a stay of 14 days or more.

^a^ Chi-square test.

^b^ Likelihood ratio.

^c^ Mann-Whitney U test.

^d^ Values from Fisher’s exact test were used.

^e^ Rifampicin sensitivity was only available for cases after Xpert was implemented in 2012.

***Source:*** Prepared by the authors from the study results, based on data from the National Tuberculosis Program database, Ministry of Health, Paramaribo, Suriname, 2019.

## DISCUSSION

This study showed that high bacillary load and age ≥ 35 years were independently associated with delayed smear conversion in PTB patients. The higher the bacillary load at start of treatment, the stronger the association (2+: AOR 1.77, 95%CI: 0.8 – 4.0; and 3+: AOR 2.34, 95%CI: 1.2 – 4.8). Several other studies have demonstrated that high bacillary load was associated with delayed clearance of sputum (8, 9, 13, 16, 17).

Regarding age group associated with delayed smear conversion, D’Souza and colleagues (9) mentioned a significant association with age ≥ 35 years. However, Caetano and colleagues (20) and Gunda and colleagues (7) found a significant association with age ≥ 50 years. Our study findings probably differ from theirs because of the differences in study populations—in the first (20), 40% of the study population was ≥ 50 years of age; in the second (7), 67% was < 34 years. In our study, 51% was 35 – 54 years and 15% was 55+ years. To the contrary, Djouma and colleagues (12) and Bisognin and colleagues (13) did not observe a significant association between older age and delayed smear conversion.

**TABLE 2.** Multivariate analysis of factors associated with smear nonconversion 2 months after tuberculosis (TB) treatment started, Suriname, 2010 – 2015 (*n* = 458)

Characteristic	Crude odds ratio	95% confidence interval	Adjusted odds ratio	95% confidence interval	*P* value
Age groups (years)					
15 – 34	1		1		
35 – 54	2.36	1.2 – 4.7	2.73	1.2 – 6.1	0.014
55+	1.55	0.6 – 4.0	2.49	1.1 – 5.9	0.039
Diabetes mellitus					
No	1		1		
Yes	0.54	0.2 – 1.4	0.47	0.2 – 1.3	0.137
HIV status					
Negative	1		1		
Positive	1.94	0.9 – 4.2	0.94	0.5 – 2.0	0.875
Bacillary load					
≤ 1+	1		1		
2+	1.05	0.5 – 2.1	1.77	0.8 – 4.0	0.171
3+	2.72	0.7 – 10.6	2.34	1.2 – 4.8	0.02
Time to start TB treatment (days)^a^					
0	1				
1 – 7	0.88	0.5 – 1.6	0.84	0.5 – 1.6	0.585
8 – 14	0.40	0.1 – 1.1	0.38	0.1 – 1.2	0.091
≥ 15	0.34	1.0 – 1.2	0.44	0.1– 1.6	0.213

^a^ This variable was categorized because it did not fulfill the monotony assumption.

***Source:*** Prepared by the authors from the study results.

The present study did not show a strong association between DM and smear nonconversion (AOR 0.47, 95%CI: 0.2 – 1.3). Other studies, however, have demonstrated a significant association between smear conversion and DM (21 – 23). This is possibly due to the prevalence of DM in the study populations, and the sample size. The prevalence of DM in our study was 9% for cases and 15% for controls, while in Shariff and colleagues (21), it was 41.3% for cases and 21.3% for controls. Though in Mi and colleagues (22) DM prevalence was 12%—similar to its prevalence in our study—those with DM were predominantly male and age > 35 years, two factors that are described in several studies to be associated with delayed smear conversion (7, 9, 15). Furthermore, Mi and colleagues did not include data on baseline smear status.

As mentioned, bacillary load is associated with delayed smear conversion. These factors might influence the association with delayed sputum smear conversion found by Mi and colleagues (22). Our study had five cases of TB with DM and delayed smear conversion at month 2. Of the five, three were female, which differs from the overrepresentation of males in the other study (22). However, additional studies have also found no association between DM and delayed smear conversion (14, 24).

HIV coinfection was not associated with delayed smear conversion in this study. The findings of other studies are somewhat contradictory regarding the influence of HIV on smear conversion 2 months after treatment initiation. Some studies (14, 19) describe a significant association between HIV coinfection and delayed sputum smear conversion; while others found no association (7, 15). Possible explanations for these differing results might be the severity of the HIV (CD4 count) and degree of adherence to ART. Unfortunately, we did not have data on CD4 levels or ART adherenc. However, a study by Nunn and colleagues described that HIV positive TB cases excrete significantly fewer organism per mL of sputum (25). If the HIV cases in our study predominantly had low bacillary load, it would not have resulted in smear nonconversion. Another possible explanation for not finding an association is that we excluded patients who died prior to receiving 2 months of treatment. These were more likely to be patients with advanced HIV/AIDS, who would have experienced delayed sputum smear conversion.

### Limitations

This study has some limitations. It is a retrospective analysis so certain known risk factors for delayed smear conversion, such as cavity on chest radiology, smoking habit, and CD4 level of HIV coinfected patients, could not be assessed because the data was not available or lacked detail. In addition, factors such as drug abuse, homelessness, being a prisoner, and smoking were excluded due to missing data (50%). Patients with an unknown outcome 2 months after treatment were excluded from the study. If these were more likely to delay smear conversion, the associated risk factors may have been underreported. For instance, patients who died before receiving 2 months of treatment were excluded and may have been more associated with HIV-seropositivity. This may have resulted in underrepresentation of HIV as a risk factor. Also, as mentioned, rifampicin sensitivity was not available for all cases. However, we believe that rifampicin resistance had no influence on study results. A recent analysis of rifampicin resistant cases (26) found that all cases had the same mutation for low-level resistance, and that after six months of treatment, the outcomes were similar to those of cases susceptible to rifampicin (26).

### Conclusions

The results of this study concur with findings of past studies indicating that higher bacillary load and age ≥ 35 year are significantly associated with delayed smear conversion. Based on these findings, the NTP of Suriname should develop strategies to improve smear conversion in the identified risk group. Patients with a higher bacillary load and age ≥ 35 should receive more focused attention to prevent tuberculosis from spreading. In addition, to better inform decision-making as well as future studies, the NTP should expand its data collection to include all risk factors for delayed smear conversion.

## Acknowledgements.

We would like to thank the staff of the National Tuberculosis Program of Suriname, especially those in the monitoring and evaluation department, as well as the nurses responsible for the data collection. Special thanks to all SORT IT facilitators and participants in the Suriname course. It was a great pleasure to learn from you. Special thanks to Zaida Yadon for her efforts to make the SORT IT project possible in Suriname. We also thank Manuel Sanchez for his support during the SORT IT course.

## Funding.

The manuscript was produced during a SORT IT training (WHO/TDR initiative) organized with PAHO support; however, no funding was received for either the study or the resulting paper.

## Author contributions.

EC, DS, DM, FP, and MS conceived the original idea, analyzed the data, interpreted the results, and wrote and reviewed the manuscript. All authors reviewed and approved the final version.

## Disclaimer.

Authors hold sole responsibility for the views expressed in the manuscript, which may not necessarily reflect the opinion or policy of the *RPSP/PAJPH* and/or PAHO.
